# The effective on intradermal acupuncture based on changes in biological specificity of acupoints for major depressive disorder: study protocol of a prospective, multicenter, randomized, controlled trial

**DOI:** 10.3389/fpsyt.2023.1183127

**Published:** 2023-06-09

**Authors:** Mingqi Tu, Xiaoting Wu, Siying Qu, Junyan Jin, Nisang Chen, Sangsang Xiong, Shuangyi Pei, Xinwei Li, Yan Shi, Hantong Hu, Xiaoyu Li, Jianqiao Fang, Xiaomei Shao

**Affiliations:** ^1^Key Laboratory for Research of Acupuncture Treatment and Transformation of Emotional Diseases, Key Laboratory of Acupuncture and Neurology of Zhejiang Province, The Third Clinical Medical College, Zhejiang Chinese Medical University, Hangzhou, China; ^2^The Third Affiliated Hospital of Zhejiang Chinese Medical University, Hangzhou, China; ^3^Tongde Hospital of Zhejiang Province, Hangzhou, China; ^4^Affiliated Hangzhou First People’s Hospital, Zhejiang University School of Medicine, Hangzhou, China

**Keywords:** major depressive disorder, intradermal acupuncture, biological specificity, acupoint, study protocol, prospective study

## Abstract

**Background:**

Antidepressants still have some side effects in treating major depressive disorder (MDD), and acupuncture therapy is a complementary therapy of research interest for MDD. Acupoints are sensitive sites for disease response and stimulation points for acupuncture treatment. Prior studies suggest that the biological specificity of acupoints is altered in physiological and pathological situations. Therefore, we hypothesize that the biological specificity of acupoints is associated with the diagnosis of MDD and that stimulating acupoints with significant biological specificity can achieve a better therapeutic effect than clinical common acupoints. This study aims to investigate the efficacy and safety of intradermal acupuncture (IA) treatment for MDD based on changes in the biological specificity of acupoints.

**Methods:**

The first part of the study will enroll 30 MDD patients and 30 healthy control (HC) participants to assess pain sensitivity and thermal specificity of MDD-related acupoints using a pressure pain threshold gauge (PTG) and infrared thermography (IRT). The potentially superior acupoints for treating MDD will be selected based on the results of PTG and IRT tests and referred to as pressure pain threshold strong response acupoints (PSA) and temperature strong response acupoints (TSA).

The second part of the study will enroll 120 eligible MDD patients randomly assigned to waiting list (WL) group, clinical common acupoint (CCA) group, TSA group, and PSA group in a 1:1:1:1 ratio. The change in the Patient Health Questionnaire-9 Items (PHQ-9), the MOS item short-form health survey (SF-36), pressure pain threshold, temperature of acupoints, and adverse effects will be observed. The outcomes of PHQ-9 and SF-36 measures will be assessed before intervention, at 3 and 6 weeks after intervention, and at a 4-week follow-up. The biological specificity of acupoint measures will be assessed before intervention and at 6 weeks after intervention. All adverse effects will be assessed.

**Discussion:**

This study will evaluate the therapeutic effect and safety of IA for MDD based on changes in the biological specificity of acupoints. It will investigate whether there is a correlation between the biological specificity of MDD-related acupoints and the diagnosis of MDD and whether stimulating strong response acupoints is superior to clinical common acupoints in the treatment of MDD. The study’s results may provide insights into the biological mechanisms of acupuncture and its potential as a complementary therapy for MDD.

**Clinical Trial Registration:**

ClinicalTrials.gov, identifier: NCT05524519.

## 1. Introduction

Major depressive disorder (MDD) is a major public health problem and one of the primary causes of disease burden worldwide because of the high lifetime prevalence, high recurrence, and high disability rate (1–3). Due to the impact of COVID-19, there is a 27.6% increase in MDD patients worldwide in 2020, with female and young adults being the most affected (4). A previous study’s results suggest that people with chronic conditions are more likely to suffer from MDD (5), which causes more stress on the patient’s life.

The antidepressant medication is currently the most common method used to treat MDD. Although this therapy has achieved good clinical efficacy, it still has certain defects, such as delayed onset of action, long treatment period, high cost, and easy relapse when the medication is discontinued (6, 7). Those side effects may reduce adherence in MDD patients. Therefore, it is imperative to optimize the clinical treatment plan for MDD.

Acupuncture, as part of traditional Chinese medical (TCM), has been shown to have advantages for MDD (8, 9). Acupuncture has been recorded to treat emotional disorders since ancient times. Moreover, it was found that intradermal acupuncture (IA) improved the changes in vagal function, blood pressure, and Beck Depression Inventory scores in MDD patients (10). Our research team conducted data mining of clinical literature on acupuncture for MDD and found that the current acupoints for MDD treatment cover 14 meridian points and extraordinary acupoints, and the most commonly used points include GV20 (Baihui), LR3 (Taichong), PC6 (Neiguan), SP6 (Sanyinjiao), GV29 (Yintang), HT7 (Shenmen), ST36 (Zusanli), etc (11). Data mining results suggest that there were noticeable discrepancies in the selection of acupoints for MDD, and there is a lack of modern scientific evidence. Therefore, this study will select acupoints by objective detection of biological specificity of acupoints and evaluate the clinical efficacy of IA on MDD in a rigorous and standardized manner.

In the area of acupoint biological specificity research, one study found that the temperature of specific acupoints such as Shenshu (BL23), Mingmen (GV4), and Baihui (GV20) was significantly lower in patients with kidney-yang deficiency MDD than in healthy individuals (12). A meta-analysis found that for high-intensity noxious stimuli, the depressed and healthy groups had similar overall pain tolerance, whereas for low-intensity stimuli, the depressed group had higher sensory thresholds and pain thresholds, suggesting that depression attenuated pain perception (13). The aforementioned studies suggest that the biological specificity of the body surface can change in MDD patients. Furthermore, a review of functional magnetic resonance imaging (fMRI) studies on acupoint specificity concluded that acupoint specificity exists, but 73.4% of these studies were conducted based on healthy subjects, 77.2% chose manual acupuncture as an intervention, and 86.1% focused on immediate efficacy (14). However, there are very few studies on the biological specificity of acupoints in patients with MDD, and a more comprehensive study of their relevance to MDD is needed.

Acupoints are specific regions through which Qi of internal organs and meridians is transported to body surface, and internal organ disorders could be reacted from the corresponding acupoints of the meridians. In recent years, more and more researchers have explored acupoints through various modern scientific techniques, such as their morphological structure (15–17), biological specificity (18), and local material basis (19). Some acupoint biological specificities contain physiological and pathological information related to their functions, and they are the external expression of the body’s vital activities (20). Enhancing research into the biological specificities of acupoints is critical for understanding the substance of meridians and acupoints and disease diagnosis and therapy.

Currently, no studies have been reported on acupuncture for MDD based on changes in the biological specificity of acupoints. Therefore, conducting this study has implications for guiding the clinical treatment of MDD with acupuncture. The well-established detection techniques of pressure pain threshold gauge (PTG) (21, 22) and infrared thermography (IRT) (23, 24) have laid a solid foundation for the correlation study of the biological specificities of acupoints. Therefore, this study will use PTG and IRT to measure pressure pain thresholds (PPT) and temperature at MDD-related acupoints, respectively, to assess 2 biological specificities, namely pain sensitivity specificity, and thermal specificity.

Based on the TCM theory that acupoints are both stimulation points for treatment and response points for disease, we propose the hypothesis that the biological specificity of acupoints is correlated with the diagnosis and treatment of diseases and that stimulating acupoints with significant biological specificity can better treat the disease than clinical common acupoints. In this study, PTG and IRT will be used to objectively present the differences in pain sensitivity specificity and thermal specificity of the MDD-related acupoints in MDD patients and healthy control (HC) participants and to screen the potentially effective acupoints for the treatment of MDD. Then, a clinical prospective randomized controlled trial will be conducted to assess the effectiveness and safety of IA for MDD based on changes in the biological specificity of acupoints.

## 2. Materials and methods

### 2.1. Aim, design and setting of the study

The overarching goal of the trial is to evaluate the therapeutic effect and safety of IA for MDD and demonstrate whether stimulating strong response acupoints is superior to clinical common acupoints in the treatment of MDD.

This study will be divided into two parts (Trial A and Trial B).

**Figure 1:**
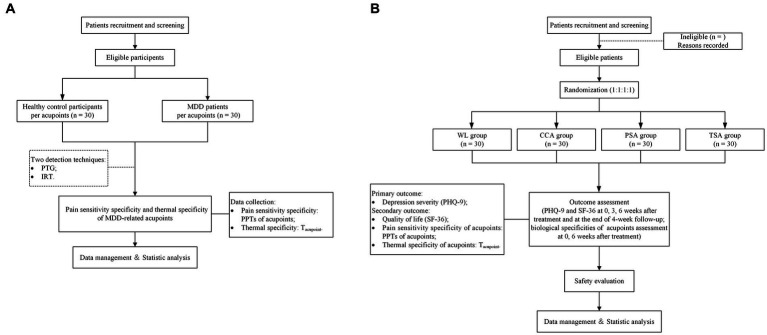
Flow chart of the study process. **(A)** Flow chart of the study process of Trial A. MDD, major depressive disorder. **(B)** Flow chart of the study process of Trial B. PTG, pressure pain threshold gauge; IRT, infrared thermography; PPT, pressure pain thresholds; T_acupoint_, temperature of the acupoint; WL, waiting list; CCA, clinical common acupoints; PSA, pressure pain threshold strong response acupoints; TSA, temperature strong response acupoints; PHQ-9, the Patient Health Questionaire-9 Items; SF-36, the MOS item short form health survey.

Trial A will adopt a controlled, assessor-blinded design, in which eligible MDD patients and HC participants will be assessed at acupoints using PTG and IRT. Figure 1A shows a flow chart of the study process. The potentially superior acupoints for the treatment of MDD will be chosen independently based on the outcomes of the PTG and IRT tests. After statistical analysis, four pressure pain threshold strong response acupoints (PSA) and four temperature strong response acupoints (TSA) will be selected, respectively. These two groups of acupoints will be used in corresponding arms in the trial B.

Trial B will be a prospective, multicenter, randomized, controlled, patient-assessor-blinded study. Eligible MDD patients will be allocated randomly to waiting list (WL) group, clinical common acupoint (CCA) group, TSA group, and PSA group in a 1:1:1:1 ratio. Figure 1B shows a flow chart of the study process, and Table 1 shows the trial schedule of enrolment, treatments, and assessments. Patients will receive 6 weeks of treatment and all patients will receive basic treatment, IA for CCA, PSA and TSA, respectively. The protocol adheres to the Standards for Reporting Interventions in Clinical Trials of Acupuncture (STRICTA) (25) (Additional file 1) and the Standard Protocol Items: Recommendations for Interventional Trials (SPIRIT) statement (26) (Additional file 2). This study is prospectively registered on ClinicalTrials.gov (NO. NCT05524519).

**Table 1:** Schedule of enrolment, treatments, and assessments.

	Study period					
	Enrolment	Allocation	Baseline	Treatment period		Follow-up period
**Timepoint**	**−t**_**1**_	**0**	**t**_**1**_ **(week 0)**	**t**_**2**_ **(week 3)**	**t**_**3**_ **(week 6)**	**t**_**4**_ **(week 10)**
**Enrolment**						
Eligibility screening	X					
Informed consent	X					
Demographic data	X					
Case data	X					
Allocation		X				
**Treatment**				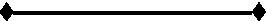		
**Assessments**						
PHQ-9			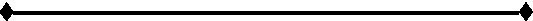			X
SF-36			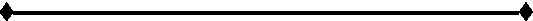			X
Pain sensitivity specificity of acupoints			X		X	
Thermal specificity of acupoints			X		X	
Safety assessment				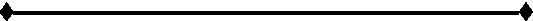		

PHQ-9, the Patient Health Questionaire-9 Items; SF-36, the MOS item short form health survey.

### 2.2. Ethical approval

The Ethics Committee of the Third Affiliated Hospital of Zhejiang Chinese Medical University granted consent (approval No: ZSLL-KY-2022-001-01-02). Each participant completed an informed consent form after receiving complete information about the experiment.

### 2.3. Study participants

To minimize the potential impact of gender and age on the biological characteristics of the 2 kinds of acupoint mentioned previously, this study will employ a gender- and age-matching strategy in the trial A, which involved the selection of HC participants and MDD patients.

### 2.4. Inclusion and exclusion criteria

The inclusion and exclusion criteria are summarized in details in Table 2.

**Table 2:** The inclusion criteria and exclusion criteria of participants of the study.

	Inclusion criteria	Exclusion criteria
HC group	(1) HC participants who could provide a recent depression screening report, and confirm they have not any cardiovascular, respiratory, digestive, urinary, hematological, endocrine, or neurological disease; (2) 18 ≤ age ≤ 60 years, male or female; (3) Participants have clear consciousness and could communicate with others normally; (4) Participants could understand the full study protocol and have high adherence. Written informed consent is signed by themselves.	(1) Participants with serious primary diseases of cardiovascular diseases, liver diseases, kidney diseases, urinary diseases, and hematological diseases; (2) Participants have a mental illness, alcohol dependence, or a history of drug abuse; (3) Pregnant or lactating participants; (4) Participants with intellectual disabilities who cannot cooperate with the questionnaire survey; (5) Participants with bleeding tendency, skin disease, allergic constitution, and allergic to adhesive tape; (6) The skin at the test site of participants has scars, hyperpigmentation, red and swollen; (7) Participants are participating in other trials.
MDD group	(1) Patients should meet the diagnostic criteria of the ICD-10 diagnostic criteria for MDD; (2) 18 ≤ age ≤ 60, male or female; (3) Participants have clear consciousness and could communicate with others normally; (4) Participants could understand the full study protocol and have high adherence. Written informed consent is signed by themselves.	(1) Participants with serious primary diseases of cardiovascular, respiratory, digestive, urinary, hematological, endocrine, neurological disease, and other serious primary diseases, and the disease cannot be effectively controlled clinically; (2) MDD caused by organic mental disorders, schizophrenia, bipolar disorder, psychoactive substances, and non-addictive substances; (3) Participants with suicidal tendencies; (4) Pregnant or lactating participants; (5) Participants with intellectual disabilities who cannot cooperate with the questionnaire survey; (6) Participants with bleeding tendency, skin disease, allergic constitution, and allergic to adhesive tape; (7) The skin at the test site of participants has scars, hyperpigmentation, red and swollen. IRT is not be performed on female subjects during their menstrual and ovulatory periods; (8) Participants are participating in other trials.

HC, Healthy control; MDD: Major depressive disorder; ICD-10, International Classification of Diseases 10th Edition.

### 2.5. Sample size estimation

Since there are no relevant references for detecting the biological characteristics of MDD-related acupoints, we referred to the literature on acupoint detection (23, 27) and planned to recruit 30 MDD patients and 30 HC participants in trial A according to the principle of minimum sample size.

In Trial B, we did not estimate the sample size based on power calculations because there was no reference to indicate how effective the use of IA for MDD might be based on changes in the biological specificity of acupoints. To meet the need for minimum sample size, we instead recruited 120 participants.

### 2.6. Recruitment

Participants will be recruited from 3 hospitals, including the Third Affiliated Hospital of Zhejiang Chinese Medical University, the Affiliated Hangzhou First People’s Hospital, and Tongde Hospital of Zhejiang Province. Posters, local newspapers, and WeChat official account promotion will be the major means of recruiting strategies for participants. The PHQ-9 will be used as the initial screening tool to identify potential MDD patients. The final diagnosis of MDD will be made by a qualified psychiatry clinician based on a comprehensive evaluation of the patient’s medical history, symptoms, and other factors that may affect their eligibility. Eligible participants will be informed about the study’s specific requirements. After signing the informed consent form, participants are invited to join the study.

### 2.7. Termination criteria and management

Subjects with one of the following conditions will be terminated: (1) serious adverse reactions that make continued participation in the trial inadvisable; (2) serious complications or deterioration conditions during the study require urgent emergency measures; (3) requesting to withdraw from the trial midway; (4) pregnancy; and (5) poor compliance and inability to follow the study protocol. When a subject has terminated, the investigator should contact the subject as soon as feasible by door-to-door, telephone appointment, or letter whenever possible. The investigator should record in detail the reason and time of termination from the study, complete the assessment items that can be completed, and those who have exceeded 1/2 course of treatment should be entered into the efficacy statistics.

### 2.8. Elimination criteria

Subjects with any of the following conditions will be eliminated: (1) not meeting the inclusion criteria but being mistakenly enrolled in the trial; (2) not following the treatment of the study protocol or having incomplete data, which affects the assessment of efficacy and safety; (3) poor compliance and withdrawing from the study on their own; (4) receiving other treatment than the intervention of this trial.

### 2.9. Randomization and allocation

Trial A is a controlled observational trial without randomization and allocation.

Trial B will use a computerized central randomization system to randomize the participants in the dynamic stratified block, which is undertaken by Zhejiang Taimei Medical Technology Co. Each center will have an independent researcher log into the computerized central randomization system and generate a randomized sequence that divides eligible participants into 4 groups (WL group, CCA group, TSA group, and PSA group) in a 1:1:1:1 ratio. The researcher will have no contact with participants, acupuncturists, outcome assessors, and data statisticians. The allocation results will be stored in the central computerized randomization system password-protected.

### 2.10. Blinding

In Trial A, detectors of biological specificity of acupoints and outcome evaluators will be blinded, while participants will not be blinded. Statistical analyses will be undertaken by third-party statisticians who will be blinded to the study protocol during the data analysis phase.

In Trial B, acupuncturists will not be blinded, considering the characteristics of acupuncture therapy. Therefore, this study will be blinded on three levels: the patient, the evaluator, and the statistician. Patients will be blinded by using a single isolated treatment room for treatment and eliminating the communication between patients for comparison purposes. Outcome evaluation will be performed by evaluators who are not aware of the allocation information. Statistical analyses will be carried out by statisticians who will not have access to allocation data. Emergency unblinding will be permitted if a serious adverse event occurs and there is a need to know what intervention was used for that patient in the study.

### 2.11. Biological specificity detection of acupoints (Trial A)

**Figure 2:**
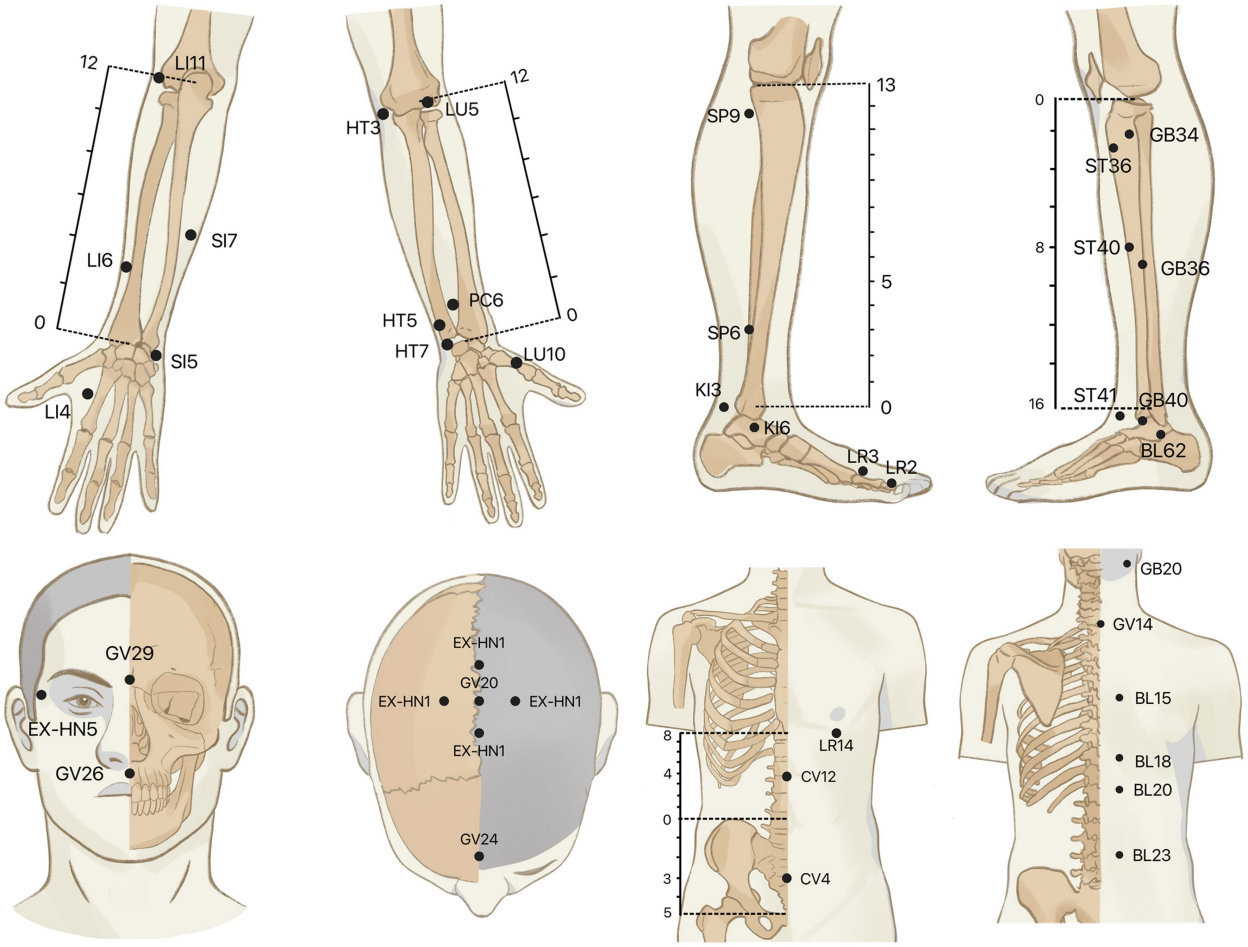
The anatomical locations of the acupoints.

To minimize the interference effects caused by confounding circumstances, all examinations will be performed almost simultaneously in the morning. A controlled condition experimental room will be adopted, with a temperature of (26 ± 1)°C, relative humidity of 40–50%, no direct sunlight and abnormal radiant source, no significant air convection, and good sound insulation. All subjects will be prohibited from drinking coffee, tea, alcohol, smoking cigarettes, or eating spicy foods on the examination day. Food and exercise will be also not allowed for at least 1 hour before the trial. After entering the experimental room, subjects will be needed to expose the test site and relax for 30 min before the formal examination to adjust to the surrounding. Throughout the exam, subjects will be asked to remain silent, breathe naturally, and relax. Before the examination, basic physiological parameters including heart rate, blood pressure, and body temperature will be taken from the subject. Based on the results of our group’s data mining of the literature related to acupuncture for MDD, 39 acupoints closely related to MDD will be selected as test sites. The IRT test will be not done on the acupoints where the hair is dense. According to the National Standard for the Name and Positioning of Acupuncture Points (GB/T 12346–2006). The anatomical locations of the acupoints are displayed in Table 3 and Figure 2.

**Table 3:** Location and indication of MDD-related acupoints.

Acupoints	Location
Head, face, and neck	
Baihui (GV20)	On the head, 5.0 cun directly above the midpoint of the anterior hairline. Or, at the midpoint of the line connecting the apexes of the two auricles.
Yintang (GV29)	At the forehead, at the midpoint between the two medial ends of the eyebrow.
Sishencong (EX-HN1)	At the vertex of the head, a group of four points, 1.0 cun, respectively, anterior, posterior and lateral to Baihui (GV 20).
Shenting (GV24)	On the head, 0.5 cun directly above the midpoint of the anterior hairline.
Shuigou (GV26)	On the face, at the junction of the superior 1/3 and middle 1/3 of the philtrum.
Taiyang (EX-HN5)	In the region of the temples, in the depression about one finger-breadth posterior to the midpoint between the lateral end of the eyebrow and the outer canthus.
Fengchi (GB20)	On the nape, below the occiput, at the level of Fengfu (GV 16), in the depression between the upper portion of m. sternocleidomastoideus and m. trapezius.
Upper limbs	
Quchi (LI11)	With the elbow flexed, the point is on the lateral end of the transverse cubital crease, at midpoint between Chize (LU 5) and the lateral epicondyle of the humerus.
Hegu (LI4)	On the dorsum of the hand, between the 1st and 2nd metacarpal bones, in the middle of the 2nd metacarpal bone on the radial side.
Pianli (LI6)	With the elbow flexed, the point is on the dorsal radial side of the forearm, on the line connecting Yangxi (LI 5) and Quchi (LI 11), 3 cun above the wrist crease
Yanggu (SI5)	On the ulnar aspect of the wrist, in the depression between the styloid process of the ulna and the triquetral bone.
Zhizheng (SI7)	On the dorsal ulnar aspect of the forearm, 5 cun above the transverse crease of the wrist, on the line connecting Yanggu (SI 5) and Xiaohai (SI 8).
Chize (LU5)	On the cubital crease, on the radial side of the tendon m. biceps brachii.
Yuji (LU10)	In the depression behind the thenar eminence of the thumb, about the midpoint of the palmar side of the thumb, on the junction of the red and white skin.
Neiguan (PC6)	On the palmar aspect of the forearm, 2 cun above the transverse crease of the wrist, on the line connecting Quze (PC 3) and Daling (PC 7), between the tendons of m. palmaris longus and m. flexor carpi radialis.
Shaohai (HT3)	When the elbow is flexed, the point is at the midpoint of the line connecting the medial end of the transverse cubital crease and the medial epicondyle of the humerus.
Tongli (HT5)	On the palmar aspect of the forearm, the point is on the radial side of the tendon m. flexor carpi ulnaris, 1.0 cun above the transverse crease of the wrist.
Shenmen (HT7)	On the wrist, at the ulnar end of the transverse crease of the writs, in the depression on the radial side of the tendon m. flexor carpi ulnaris.
Lower limbs	
Zusanli (ST36)	On the anterior aspect of the lower leg, 3 cun below Dubi (ST 35), one finger-breadth (middle finger) from the anterior crest of the tibia.
Fenglong (ST40)	On the anterior aspect of the lower leg, 8 cun superior to the external malleolus, lateral to Tiaokou (ST 38), two finger-breadth (middle finger) from the anterior crest of the tibia.
Jiexi (ST41)	At the junction of the dorsum of the foot and the lower leg. in the depression at the midpoint of the transverse crease of the ankle between the tendons m. extensor hallucis longus and digitorum longus.
Yanglingquan (GB34)	On the lateral aspect of the lower leg, in the depression anterior and inferior to the head of the fibula.
Waiqiu (GB36)	On the lateral aspect of the lower leg, 7 cun above the tip of the external malleolus, on the anterior border of the fibula, at the level of Yangjiao (GB 35).
Qiuxu (GB40)	On the foot, anterior and inferior to the external malleolus, in the depression on the lateral side of the tendon of m. extensor digitorum longus.
Shenmai (BL62)	In the depression directly below the external malleolus.
Sanyinjiao (SP6)	On the medial aspect of the lower leg, 3 cun above the medial malleolus, on the posterior border of the medial aspect of the tibia.
Yinlingquan (SP9)	On the medial aspect of the lower leg, in the depression of the lower border of the medial condyle of the tibia.
Taichong (LR3)	On the dorsum of the foot, in the depression proximal to the 1st metatarsal space.
Taixi (KI3)	On the medial aspect of the foot, posterior to the medial malleolus, in the depression between the tip of the medial malleolus and tendo calcaneus.
Zhaohai (KI6)	On the medial aspect of the foot, in the depression below the tip of the medial malleolus.
Xingjian (LR2)	On the dorsum of the foot, proximal to the margin of the web between the 1st and 2nd toes, at the junction of the red and white skin.
Back and lumbar	
Dazhui (GV14)	On the posterior median line, in the depression below the spinous process of the 7th cervical vertebra.
Xinshu (BL15)	On the back, 1.5 cun lateral to the lower border of the spinous process of the 5th thoracic vertebra.
Ganshu (BL18)	On the back, 1.5 cun lateral to the lower border of the spinous process of the 9th thoracic vertebra.
Pishu (BL20)	On the back, 1.5 cun lateral to the lower border of the spinous process of the 11th thoracic vertebra.
Shenshu (BL23)	On the back, 1.5 cun lateral to the lower border of the spinous process of the 2nd lumbar vertebra.
Chest and abdomen	
Guanyuan (CV4)	On the anterior median line of the lower abdomen, 3 cun below the umbilicus.
Zhongwan (CV12)	On the anterior median line of the upper abdomen, 4.0 cun above the umbilicus.
Qimen (LR14)	On the chest, directly below the nipple, in the 6th intercostal space, 4 cun lateral to the anterior midline.

MDD, Major depressive disorder.

#### 2.11.1. PTG

The FDX25 handheld pressure pain gauge will be used to detect the PPT of acupoints. The probe will be positioned vertically on the measurement acupoint, pressure will be applied slowly and steadily, and pressure will be withdrawn as soon as the patient feels pain. The PPT of the acupoint will be displayed on the gauge. Repeat the procedure 3 times at 5 min intervals.

The data will be entered into Excel. The basic PPT of the acupoint will be determined as the average of the 3 times PPT values. The top 4 acupoints most associated with MDD in PTG test will be selected as PSA by logistic regression analysis.

#### 2.11.2. IRT

An infrared thermography camera (NEC InfRec R450, Avio Infrared Technologies Co., Ltd., Tokyo) with measuring wavelengths of 8–14 um, a temperature resolution of 0.025°C, and a resolution of 640 × 480 pixels, will be used to record thermal images to assess the heat radiation properties of the acupuncture points. By adjusting the height and angle of the machine, the subject’s measured sites will be on the camera screen distinctly. One researcher will use a cotton swab to mark the tested acupoints, and another researcher will operate the electronic cursor of the camera to the corresponding acupoint localization and record the coordinates. Turn off the lights and focus on the camera, then start recording the thermal images. Using the interval shooting mode, 1 thermal image will be taken automatically every 10s, and 1 min continuously, a total of 6 thermal images will be taken. The subject will be instructed to remain still during the measurement.

The InfRec Analyzer NS9500 (Avio Infrared Technologies Co., Ltd., Tokyo) will analyze all thermal images, displaying different temperatures in different colors and selecting the region where the temperature will be measured. The temperature data collecting parameter of the region of interest (ROI) will be set up as a circular area within a radius of 5 mm from the acupoint, which will be the same size as an acupoint in this study. TROI will be used as the average temperature of the ROI in 1 thermal image. The average TROI in the 6 thermal images will be calculated as the temperature of the acupoint (T_acupoint_). The top 4 acupoints most associated with MDD in IRT test will be selected as TSA by logistic regression analysis.

### 2.12. Intervention (Trial B)

All patients will receive basic treatment: (1) health education: Properly explain the causes and precautions of the patient and instruct the patient to rest regularly. In addition, care should be enhanced to avoid accidents; (2) the basic therapeutic drug is the use of one or more antidepressants, such as oral 5-hydroxytryptamine reuptake inhibitors, dual inhibitors of pentraxin and norepinephrine reuptake, noradrenergic and specific pentraxinergic antidepressants, pentraxin receptor balance antagonists, tricyclic antidepressants, etc. If there is significant sleep disturbance, benzodiazepines may be added temporarily. The dose and frequency of the medication will be adjusted by the specialist. The treatment course of all groups will be up to 6 weeks and a 4-week follow-up.

**Figure 3:**
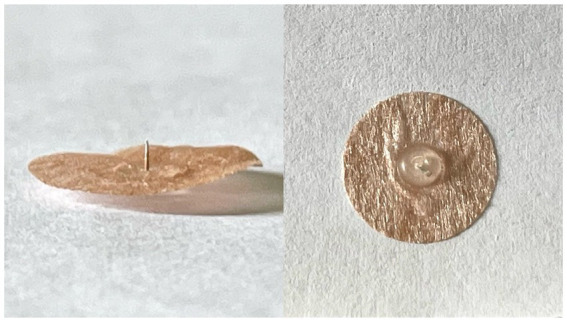
The appearance of intradermal needle.

This study will use the intradermal needle (SEIRIN Co., Japan) as an acupuncture intervention, and its appearance is shown in Figure 3. All acupoints will be taken bilaterally.

#### 2.12.1. WL group

Patients in this group will not receive IA treatment during the study period. When the study is over, they will receive 6 weeks of the same IA treatment as in the CCA group.

#### 2.12.2. CCA group

LR3 (Taichong), PC6 (Neiguan), SP6 (Sanyinjiao) and HT7 (Shengmen) will be stimulated in this group. PC6 and HT7 will use φ0.20*1.2 mm needle, as well as LR3 and SP6 will use φ0.20*1.5 m needle. After routinely disinfecting the skin around the selected acupoints, press the needle to insert the acupoint vertically and retain it in the skin. After the intervention, the needle will be retained for 72 h with a day of rest after removal. Then the next treatment will be performed. During needle retention, participants will be instructed to press the acupoints 3–4 times a day for about 1 min each time, with the amount of stimulation as much as the patient can tolerate, at an interval of about 4 h. A total of 10 treatment sessions will be performed for 6 weeks.

#### 2.12.3. PSA group

PSA selected in Trial A will be stimulated. According to the location of the acupoint, φ0.20*1.5 m needles are selected for thick muscles and φ0.20*1.2 mm needles are selected for shallow muscles. The operation, and treatment sessions will be the same as the CCA group.

#### 2.12.4. TSA group

TSA selected in Trial A will be stimulated. The needle, operation, and treatment sessions will be the same as the PSA group.

### 2.13. Outcome Measures

#### 2.13.1. Trial A

(1) Pain sensitivity specificity: PPT of acupoints;

(2) Thermal specificity: temperature of acupoints.

#### 2.13.2. Trial B

(1) Primary outcome measures: change in the PHQ-9. The PHQ-9 scale is widely used in clinics to assess MDD symptoms and evaluate therapy response (28, 29). There are nine questions on the PHQ-9 scale, each with a score of 0–3 (none: 0; a few days: 1; more than half of the time: 2; almost every day: 3), for a total score of 0–27. PHQ-9 has been demonstrated to be reliable in studies because of its high sensitivity and specificity (30). The accuracy and ability of the PHQ-9 to detect depression results over time have been proven in studies, and it can be utilized as a valid prognostic indicator (31).

(2) Secondary outcome measures: (1) Change in the SF-36: the MOS 36-item Short Form health survey (SF-36) (32) consists of 36 items that provide a comprehensive assessment of the patient’s physical condition and emotional perception in 8 dimensions: physical function (PF), role physical (RP), body pain (BP), general health (GH), vitality (VT), social function (SF), role emotional (RE), and mental health (MH), (2) Pain sensitivity specificity: the PPT change of acupoints, (3) Thermal specificity: the T_acupoint_ change of acupoints.

The PHQ-9 and SF-36 will be assessed before intervention, at 3, 6 weeks after intervention and at the end of 4-week follow-up; biological specificities of acupoints will be assessed before intervention, at 6 weeks after treatment.

### 2.14. Safety Assessment

The safety assessment indicator is the frequency of adverse events (AEs) during treatment in the trial. AEs will be recorded, such as bleeding, hematoma, dizziness, pain at the needle site, and increased blood pressure caused by acupuncture. AEs related to drug administration mainly include drug allergy, gastrointestinal disorders, neurological symptoms such as headache or sleep abnormalities, etc. Whether or not the AEs are related to the treatment method of this study, they should be recorded in detail, including the date of occurrence, duration, severity, and treatment measures of the AEs, etc. Investigators will assess the relevance of AEs to treatment.

When AEs occur, patients will be documented and treated promptly and appropriately until they return to fully normal. For serious AEs, investigators should immediately report to the principal investigator and the ethics committee, who will decide whether the patients should be withdrawn.

### 2.15. Quality Control

Before the trial, all investigators will receive training on the study protocol and various standard operating procedures (SOP) according to their different roles, to improve internal observational consistency and interobserver consistency among investigators, ensure the reliability of clinical study findings, and reduce the risk of bias. All acupuncture operations will be performed by acupuncturists who are licensed physicians and have been independently practicing acupuncture for at least 3 years and require training in the clinical standard practice of this study. Western medicine prescriptions will be performed by experienced, uniformly trained specialists. All outcome assessors will participate in standard outcome assessment practice training and uniformly complete case report forms (CRFs). Throughout the trial, all study data will be collected on CRFs and entered into the electronic data capture (EDC) system by independent investigators promptly. In addition, to ensure data accuracy, each center will arrange for another independent investigator to verify the EDC system data against the CRF. Mid-trial withdrawals or drop-outs will be documented in detail.

Under the direction of the Data Safety Monitoring Committee, a data safety monitoring committee will be formed. This committee’s staff will be in charge of data monitoring and will have the authority to reveal blinded data. They will also verify the authenticity between the raw and recorded data. In addition, the investigators will strictly implement a monthly clinical study quality check and quarterly multi-center clinical study quality monitoring, and discuss difficulties and best solutions that arise throughout the trial to control inter-center bias.

All treatment expenses and outcome assessments will be provided free of charge to participants to encourage recruitment and compliance.

### 2.16. Statistical Analysis

Statistical analyses will be performed by a third-party statistician not involved in the pre-test using the statistical software SPSS 25.0 (SPSS Inc., Chicago, IL, United States). It will be expressed as counts and percentages for categorical variables. Continuous variables in normal distribution will be expressed as mean ± standard deviation, while skewed data will be expressed as median and interquartile range. The chi-Squared test will be used to compare group differences in dichotomous variables with baseline characteristics such as gender and age. Logistics regression analysis will be used to screen potentially superior acupoints. To analyze changes in continuous variables at multiple time intervals before and after the intervention, normally distributed data will be compared within and between groups using repeated measures analysis of variance (ANOVA). For within- or between-group comparisons, non-parametric test will be utilized for skewed distribution data. Statistical significance is defined as a *p* value <0.05.

The intention-to-treat approach will be adopted. Missing data (imputation of missing cases by regression and imputation from the last observation) and non-compliance will be subjected to sensitivity studies (analysis by protocol).

## 3. Discussion

In TCM theories, acupoints are both response points to disease and stimulation points for treating disease. Therefore, we hypothesize that the biological specificity of acupoints is correlated with the diagnosis and treatment of diseases and that stimulating acupoints with significant biological specificity can better treat the disease than clinical common acupoints. There are no clinical studies based on changes in the biological specificity of acupoints for MDD. Many clinical studies have shown that acupuncture is effective in improving the symptoms of MDD. However, most studies have been based on the empirical selection of acupoints, lacking modern scientific support. The altered pain sensitivity specificity and the thermal sensitivity of acupoints are two of the main forms of altered biological specificities of acupoints in pathological states (33). PTG is a safe and noninvasive approach for converting subjective pain experience into objective values, and PPT is one of the traditional methods to measure pain sensation (34). IRT is a new medical imaging technique based on infrared radiation emitted from the surface of an object to quantify and visualize the temperature distribution on the object’s surface, which has the advantage of being non-contact and non-invasive (35). Therefore, the first step of this study is to observe the pain sensitivity specificity and the thermal sensitivity of MDD-related acupoints based on acupoint biological specificity detection techniques and further explore the correlation between the biological specificity of acupoints and diseases. By testing the biological specificity of acupoints in the first step of the study, we will screen the strongly responsive acupoints as possible superior acupoints for the treatment of MDD. In a subsequent prospective, multicenter, randomized, controlled trial, we will compare the efficacy of WL group, CCA group, PSA group and TSA group for MDD and changes in the biological specificity of acupoints before and after treatment. The efficacy of an acupoint selection approach based on altered acupoint biology will be validated by this RCT study. It is also hoped that modern tests that can be used to guide the clinical selection of acupoints for the treatment of MDD can be screened.

Although traditional acupuncture has some advantages in improving MDD, it still has some limitations, such as time-consuming and some people having a fear of acupuncture. IA is a special acupuncture therapy that provide soft continuous stimulation of targeted acupoints for several days. Comparing to traditional acupuncture, IA is safer, more convenient and painless. Previous studies have found no significant difference between shallow acupuncture, which is similar to IA to some extent, and conventional acupuncture in activation of the cerebral cortex associated with MDD (36, 37). Moreover, a preclinical study has shown that IA can improve the behavior of depressed mice, increase their serum 5-HT levels and effectively regulate the function of the hypothalamic pituitary adrenal axis (38). Therefore, in this study, IA will be used as an acupuncture intervention to observe the clinical efficacy of IA for MDD based on the altered biological specificity of acupoints.

Guideline for primary care of MDD (2021) recommends a medication trial period of 4–6 weeks for patients undergoing depression treatment (39). Acupuncture treatments for depressive disorders typically last 4 to 6 weeks, according to the most recent expert consensus of integrated traditional Chinese and Western medicine for depression disorder (40). There are many previous studies of acupuncture for MDD that also chose 6 weeks as the endpoint (41–43). As a result, we chose 6 weeks as the time point for evaluation of the final treatment. This supports the validity of our study and ensures that our findings are in line with current best practices in the treatment of MDD.

Despite this study having many strengths and highlights, it does have some limitations. First, although this study attempts to limit the influence of exogenous and endogenous factors on acupuncture point recognition by controlling the ambient temperature or humidity and requiring patients to rest enough before doing the test, there may be some errors in the two detection techniques. In addition, to minimize measurement errors, we will provide uniform training for the testing staff before the formal test. Second, due to time and financial constraints, large sample size and long-term follow-up will be not possible, so the sample size of this study will be small with a short follow-up period. However, the results of this study may still provide evidence for the feasibility of this trial design and provide basic data for future full-scale trials. We hope to expand the sample size and extend the follow-up period in the future to assess the long-term treatment effects of snap for MDD. Third, the nature of the IA treatment method makes it difficult to blind the IA operators in our study. To minimize the subjective influence, our study outcome assessment and statistical analysis will be performed by a third party who is unaware of the subgroup. Fourth, we only set up a medicine group without setup a sham acupuncture control group. Although we do not set up a sham IA group, thereby failing to exclude a possible placebo effect of IA. However, we believe that if there is a placebo effect, it is part of the IA effect.

## 4. Conclusion

This protocol outlines the methodology of a prospective, multicenter, randomized, controlled trial investigating the therapeutic efficacy and safety of IA for MDD, based on changes in the biological specificity of acupoints. The study aims to explore the correlation between MDD-related acupoint specificity and MDD diagnosis, as well as the effectiveness of stimulating strong response acupoints compared to clinical common acupoints. The study’s results may provide valuable insights into the biological mechanisms of acupuncture and its potential as a complementary therapy for MDD.

## Ethics statement

The studies involving human participants were reviewed and approved by the Ethics Committee of the Third Affiliated Hospital of Zhejiang University of Traditional Chinese Medicine. The patients/participants provided their written informed consent to participate in this study.

## Author contributions

XS is responsible for this study. JF and XS designed the trial protocol, while MT wrote the manuscript. The manuscript was revised by HH, XW and XL. MT, XW, SQ, and JJ devised a plan for data analysis. NC and SX took part in the recruiting process. XS, MT, XW, XL, and YS were involved in developing the main documents regarding ethics approval and data assurance that constitute the basis of this manuscript. All authors contributed to the article and approved the submitted version.

## Funding

The work was financially supported by the Zhejiang Traditional Chinese Medicine Administration (2022ZX010).

## Acknowledgments

The authors appreciate the support of all participants who have been or will be included in this study.

## Conflict of interest

The authors declare that the research was conducted in the absence of any commercial or financial relationships that could be construed as a potential conflict of interest.

## Publisher’s note

All claims expressed in this article are solely those of the authors and do not necessarily represent those of their affiliated organizations, or those of the publisher, the editors and the reviewers. Any product that may be evaluated in this article, or claim that may be made by its manufacturer, is not guaranteed or endorsed by the publisher.

## Supplementary material

SUPPLEMENTARY TABLE S1 STRICTA 2010 checklist of information to include when reporting interventions in a clinical trial of acupuncture.

SUPPLEMENTARY TABLE S2 SPIRIT 2013 Checklist: Recommended items to address in a clinical trial protocol and related documents.

## Glossary

MDD: Major depressive disorder
TCM: Traditional Chinese medical
IA: Intradermal acupuncture
fMRI: functional magnetic resonance imaging
HC: Healthy control
PTG: Pressure pain threshold gauge
IRT: Infrared thermography
WL: Waiting list
CCA: clinical common acupoint
STRICTA: Standards for Reporting Interventions in Clinical Trials of Acupuncture
SPIRIT: Standard Protocol Items Recommendations for Interventional Trials Statement
PSA: Pressure pain threshold strong response acupoints
TSA: Temperature strong response acupoints
PPT: Pressure pain threshold
ROI: Region of interest
T_acupoint_: Temperature of the acupoint
PHQ-9: Patient Health Questionaire-9 Items
SF-36: the MOS item short form health survey
PF: physical function
RP: role physical
BP: body pain
GH: general health
VT: vitality
SF: social function
RE: role emotional
MH: mental health
AEs: Adverse events
SOP: Standard operating procedures
CRFs: Case report forms
EDC: Electronic data capture
ANOVA: Analysis of variance
